# Not on the same wavelength? How autistic traits influence cooperation: evidence from fNIRS hyperscanning

**DOI:** 10.3389/fpsyt.2024.1514682

**Published:** 2024-12-23

**Authors:** Kaiyun Li, Bang Du, Xue Guan, Liu Chen, Mingxue Wang, Gongxiang Chen, Fanlu Jia, Xiaoqing Jiang

**Affiliations:** ^1^ School of Education and Psychology, University of Jinan, Jinan, China; ^2^ Information Science and Engineering, University of Jinan, Jinan, China

**Keywords:** autistic traits, cooperation, empathy, interpersonal brain synchronization, fNIRS

## Abstract

**Background:**

Individuals with high autistic traits exhibit characteristics like those of individuals with autism, including impairments in sociability and communication skills. Whether individuals with high autistic traits exhibit less cooperation remains debated.

**Methods:**

This study employed the prisoner’s dilemma game (PDG) to measure cooperation in 56 dyads, including 27 with high-low (HL) autistic traits and 29 with low-low (LL) autistic traits, using functional near-infrared spectroscopy (fNIRS) hyperscanning technique. Cognitive and emotional empathy were also measured.

**Results:**

Individuals with high autistic traits had a lower unilateral cooperation rate than did those with low autistic traits; The HL autistic dyads exhibited a lower mutual cooperation rate and reduced interpersonal brain synchronization (IBS) in the right inferior parietal lobule (r-IPL) and right temporoparietal junction (r-TPJ) compared with the LL autistic dyads; Individuals with high autistic traits had significantly lower cognitive empathy scores than did those with low autistic traits, and the cognitive empathy scores had a marginally significant positive correlation with the unilateral cooperation rate and a marginally significant negative correlation with the activation of the right inferior frontal gyrus (r-IFG); Emotional empathy scores did not significantly differ between the high and low autistic groups, and there was a significant positive correlation between emotional empathy scores and the activation of the r-IFG in individuals with high autistic traits.

**Conclusion:**

This study revealed abnormal cooperation in individuals with high autistic traits from unilateral and mutual behavior neural perspectives, potentially linked to a disability of cognitive empathy.

## Introduction

Autism is a neurodevelopmental condition characterized by difficulties in social communication, restricted interests, and stereotyped behavior (1). Initially, autism diagnoses were simply categorized as having or not having the condition. However, as research has progressed, the concept of an “autistic continuum” has emerged. This continuum suggests that everyone has some degree of autistic traits, with everyone finding their place along this spectrum (2). Autistic traits are prevalent in the general population. Although individuals with high levels of these traits may not meet the clinical criteria for autism, their symptoms can resemble those of autism patients. These symptoms include difficulties in social skills, lower empathy, and reduced cognitive flexibility (3, 4). Cooperation is essential for pursuing common interests and achieving success, as well as a key element in sustainable social development (5). Participating in cooperative activities fosters meaningful social interactions and friendships, reducing the likelihood of social withdrawal and isolation (6); therefore, cooperation is very important for the social adaptation of individuals with autism and those with high autistic traits.

Some studies suggest that individuals with autism often perform worse on cooperative tasks than their non-autistic peers do (7, 8). For example, Liebal et al. (8) assessed cooperative behavior in autistic children through four cooperative tasks with adults and reported that autistic children were significantly less successful than non-autistic children were. Similarly, Colombi et al. (7) explored the cooperative performance of autistic children through four non-verbal cooperative tasks and reported that their cooperation scores were significantly lower than those of the control group. However, other studies have reported no significant differences in cooperation between autistic and non-autistic individuals. A comparison of cooperation levels between autistic and non-autistic children using the classic prisoner’s dilemma game (PDG) revealed no significant differences (9, 10). These inconsistent results may stem from variations in the severity of autism, the limited cooperation abilities of autistic participants, and differences in the cooperative tasks used. More importantly, these studies compared the cooperation performance of autistic children to that of non-autistic children without focusing on the social interactivity aspect of cooperation. Cooperation involves interactions between at least two individuals with the aim of maintaining harmonious relationships and common interests. Without interpersonal interactions, cooperation cannot occur; therefore, social interactivity is a core feature of cooperation (9–11). Given that autism exists on a continuum and that autistic individuals often have limited cooperation abilities, studying individuals with high autistic traits can provide crucial evidence for clinical research. However, only Craig et al. (12) explored the relationships among autistic traits, theory of mind (ToM), and cooperation using a socioeconomic game. They reported that individuals with high autistic traits had difficulty cooperating with ToM agents but performed better when cooperating with fixed strategy agents, although they needed more time to express cooperative intentions. Nevertheless, the cooperation measures used in this study also failed to reflect the social interactivity aspect of cooperation. In summary, previous research on the cooperation of both clinically diagnosed autistic patients and those with high autistic traits has not examined mechanisms from the perspective of social interactivity in the cooperation process.

In recent years, researchers have increasingly adopted functional near-infrared spectroscopy (fNIRS) hyperscanning technology to study social interactions between individuals. Unlike functional magnetic resonance imaging (fMRI), which suffers from low ecological validity, and electroencephalography (EEG), which has limited spatial resolution and is vulnerable to motion artifacts, fNIRS stands out for its resistance to motion artifacts and its high ecological validity (13). As a result, fNIRS was selected as the preferred method for hyperscanning research in this study. Interaction quality is measured using interpersonal brain synchronization (IBS) as an indicator (14–17). IBS refers to the synchronized changes in brain activity between two or more individuals during social activities or joint tasks (18). Numerous studies have demonstrated that IBS is a reliable indicator of social interaction quality (14, 19–21). Studies in individuals without autism using fNIRS hyperscanning technology have shown that during cooperation, IBS occurs between the frontal regions and the mirror neuron system-related brain areas of cooperating individuals (15, 22, 23). For example, Pan et al. (15) investigated brain activity in the right frontal–parietal regions of male–female pairs (partners, friends, and strangers) while they were performing a cooperative task. Compared with the friend and stranger groups, the partner group exhibited better cooperative behavior and increased IBS in the right superior frontal cortex. Zhang et al. (23) employed the classic cooperative tangram puzzle task to study brain activity in friend and stranger groups during joint and divided cooperation and reported that the friend group exhibited stronger IBS in the brain regions related to the mirror neuron system than did the stranger group. These studies suggest that, in the context of cooperation, IBS reflects participants’ cognitive and emotional alignment, coordination, and mutual understanding, highlighting how effectively they work together and communicate to achieve common goals. Recently, Peng et al. (24) studied the social communication abilities of 64 pairs of university students with high-high (HH), low-low (LL), and high-low (HL) autistic traits. They reported that the HL group exhibited lower IBS during social communication interactions, whereas the HH group exhibited increased IBS during social communication. This finding suggests that individuals with high autistic traits possess effective communication abilities, depending on their interaction partners. However, it is still unknown whether individuals with high autistic traits can normally interact with those with low autistic traits during cooperation, especially in terms of IBS. Therefore, in this study, fNIRS hyperscanning technology was used to explore the cooperation of individuals with high and low autistic traits to further understand the cooperation of individuals with high autistic traits.

Many factors can influence cooperation, and empathy is widely regarded as a key factor (25–27). Empathy consists of two fundamental components: cognitive and emotional empathy (28). Cognitive empathy refers to recognizing others’ emotions and thoughts, including perspective-taking and ToM capabilities. Emotional empathy involves perceiving and sharing others’ emotional states (29–31). Sanchez (26) posits that the ability to cooperate relies on understanding others’ psychological and emotional states. Some studies have identified deficits in both cognitive and emotional empathy in individuals with high autistic traits (32–35). For example, Zhang et al. (36) recently reported significant negative correlations between autistic traits and both cognitive and emotional empathy. These findings support the mind blindness hypothesis proposed by Baron-Cohen et al. (37), suggesting that individuals with high autistic traits have impaired empathy, making it difficult for them to understand others’ feelings, thoughts, and beliefs. However, Smith (38) proposed the empathy imbalance theory, suggesting that individuals with high autistic traits have deficits in cognitive empathy but intact emotional empathy. This theory has received support from several studies (31, 39). Le et al. (39) reported that individuals with high autistic traits exhibited significant deficits in cognitive empathy but not in emotional empathy. Given the inconsistent relationship between empathy and cooperation reported in studies of individuals with autistic traits, this study further analyzed the relationship between cognitive and emotional empathy and the cooperation of individuals with autistic traits.

In summary, this study utilized fNIRS hyperscanning technology to investigate the effects of autistic traits on cooperation and their relationship with empathy. The participants were selected according to the Autism Spectrum Quotient (AQ) and divided into two paired groups (HL and LL groups). This study focused on the inferior parietal lobule (IPL), temporoparietal junction (TPJ), and inferior frontal gyrus (IFG) as regions of interest for measuring IBS. IPL is linked to perspective-taking and self-other differentiation (15, 40). The TPJ plays a crucial role in considering the mental states of others (41, 42), while the IFG is involved in understanding others’ intentions and empathizing with their emotions (15, 40). Yang et al. (17) demonstrated increased IBS in the bilateral IPL during cooperative situations using a joint Simon task. Similarly, Zhou et al. (43) found stronger IBS in the right TPJ when participants received instructions from a navigator rather than a computer during a social navigation task. In a related study, Zhou et al. (44) observed stronger IBS in the right TPJ during successful cooperative jigsaw puzzle solving. Additionally, Liu et al. (40) showed that activation of the right IFG in the builder increased in response to supportive actions from the cooperator in a turn-taking game. These studies demonstrate that these brain regions are closely linked to cooperation and play key roles in social interaction (15, 17, 40–49). On the basis of previous research on the cooperation of individuals with autism traits and those with high autistic traits, this study proposes the following hypotheses: (1) high autistic traits lead to reduced cooperation rates, with individuals who have high autistic traits scoring lower in cognitive or emotional empathy; (2) individuals with high autistic traits may exhibit abnormal activation patterns in the IFG, IPL, and TPJ, which correlate with their cognitive and emotional empathy abilities; and (3) the HL group will have a lower IBS in the regions of interest (IFG, TPJ, and IPL) than the LL group.

## Methods

### Participants

The sample size was estimated using G*Power 3.1.9.7, which indicated that a minimum of 34 dyads (17 per group) was required, based on the following parameters: medium effect size (0.25), 1 - *β* = 0.80, and *α* = 0.05 (50). We distributed 503 AQ questionnaires and identified participants with varying levels of autistic traits according to the following criteria: the top 5% of high AQ scores were classified as high autistic traits, and scores below the mean minus one standard deviation were classified as low autistic traits (51, 52). Participants with high autistic traits (score ≥ 30) and low autistic traits (score ≤ 16) were included in the study. The participants were randomly assigned to two groups based on their autistic traits: HL and LL dyads. Sex was consistent within each pair of groups. Initially, there were 30 HL dyads and 31 LL dyads. However, some participants did not answer carefully, and excessive hair prevented effective fNIRS data collection. Consequently, data from 112 participants (27 HL dyads and 29 LL dyads, 27 individuals with high autistic traits and 85 individuals with low autistic traits) were included in the analysis. Detailed information is provided in **Table 1**. All experiments conducted in this study were approved by the local ethical committee of the university. Written consent was obtained from participants in this study.

**Table 1:** Participants’ demographic characteristics.

				Age (in years)		Sex	
Group		Autistic traits	*n*	Range	*M ± SD*	Male	Female
High-low	High		27	18-22	19.36 ± 0.89	17	10
	Low		27	18-22	19.46 ± 0.94	17	10
Low-low	Low		29	18-22	19.42 ± 0.86	13	16
	Low		29	18-22	19.15 ± 0.71	13	16

### Experimental materials

#### Autism spectrum quotient

The AQ was used to assess the main symptoms and behavioral patterns of autism (51). The total AQ score ranges from 0 to 50, with higher scores indicating more pronounced autistic traits. Lin (53) translated the Taiwanese version of the AQ questionnaire into simplified Chinese and validated it in Chinese college students. The current study used this version.

#### The prisoner’s dilemma game

The cooperative task utilized the PDG. At the start of the task, the experimenter provided instructions and presented the participants with a sample payoff matrix, which explained how to respond. During this multi-round game, participants viewed the payoff matrix and a prompt, asking them to press “D” or “J” on the keyboard to cooperate or “F” or “K” to defect. The payoff matrix displayed the scores each player would receive according to their combination of moves. If both participants chose to cooperate, each earned 2 points. If both participants chose to defect, each earned 1 point. If one participant cooperated while the other defected, the cooperator scored 0 points, and the defector scored 3 points. Individually, choosing to defect yields a higher expected payoff than choosing to cooperate. However, from a collective standpoint, cooperation leads to a higher total payoff than defection does (54).

#### Cognitive empathy task

The cognitive empathy task was based on the study by Dodell-Feder et al. (55) and included both experimental and neutral conditions. In the experimental condition, a false-belief task was used to evaluate participants’ cognitive empathy abilities. For example, participants were presented with the following text: “Jenny places chocolate in a cupboard and then leaves. Alan moves the chocolate from the cupboard to the refrigerator. Half an hour later, Jenny returns to the room.” The participants are then asked to judge whether the statement “Jenny believes she can find the chocolate in the cupboard” is correct. In the neutral condition, participants perform a regular statement judgment task to assess their performance on non-empathy-related materials. They are given the following text: “A traffic camera captures an image of a black car accelerating through a red light. Shortly after, the car is painted red, and its license plate is changed.” The participants must then judge whether the statement “According to the traffic camera, the car is black” is correct.

#### Emotional empathy task

The emotional empathy task was based on the Multidimensional Empathy Test and the experimental paradigm of Oliver et al. (56). This task included both experimental and neutral conditions. In the experimental condition, participants rated the intensity of their empathic response to the emotional states of individuals depicted in images. To reduce the influence of cognitive empathy, the emotional states were labeled on the images. In the neutral condition, participants simply estimated the ages of the individuals shown in the images.

### Experimental design

A two-factor mixed experimental design was employed. For individual behavior results and brain activation intensity, the two independent variables were autistic traits (high vs. low) and decision outcome (unilateral cooperation vs. unilateral defection). For dyadic behavior results and IBS, the two independent variables were group (HL vs. LL) and decision outcome (mutual cooperation vs. mutual defection). The dependent variables were divided into behavioral and neural indicators. The behavioral indicators included the probability of unilateral cooperation/defection and mutual cooperation/defection in the PDG, the accuracy and reaction time in the cognitive empathy task, and the ratings and reaction time in the emotional empathy task. Neural indicators included the activation value (*β*) of individual brains and IBS during corresponding behavioral responses.

### Procedure

**Figure 1:**
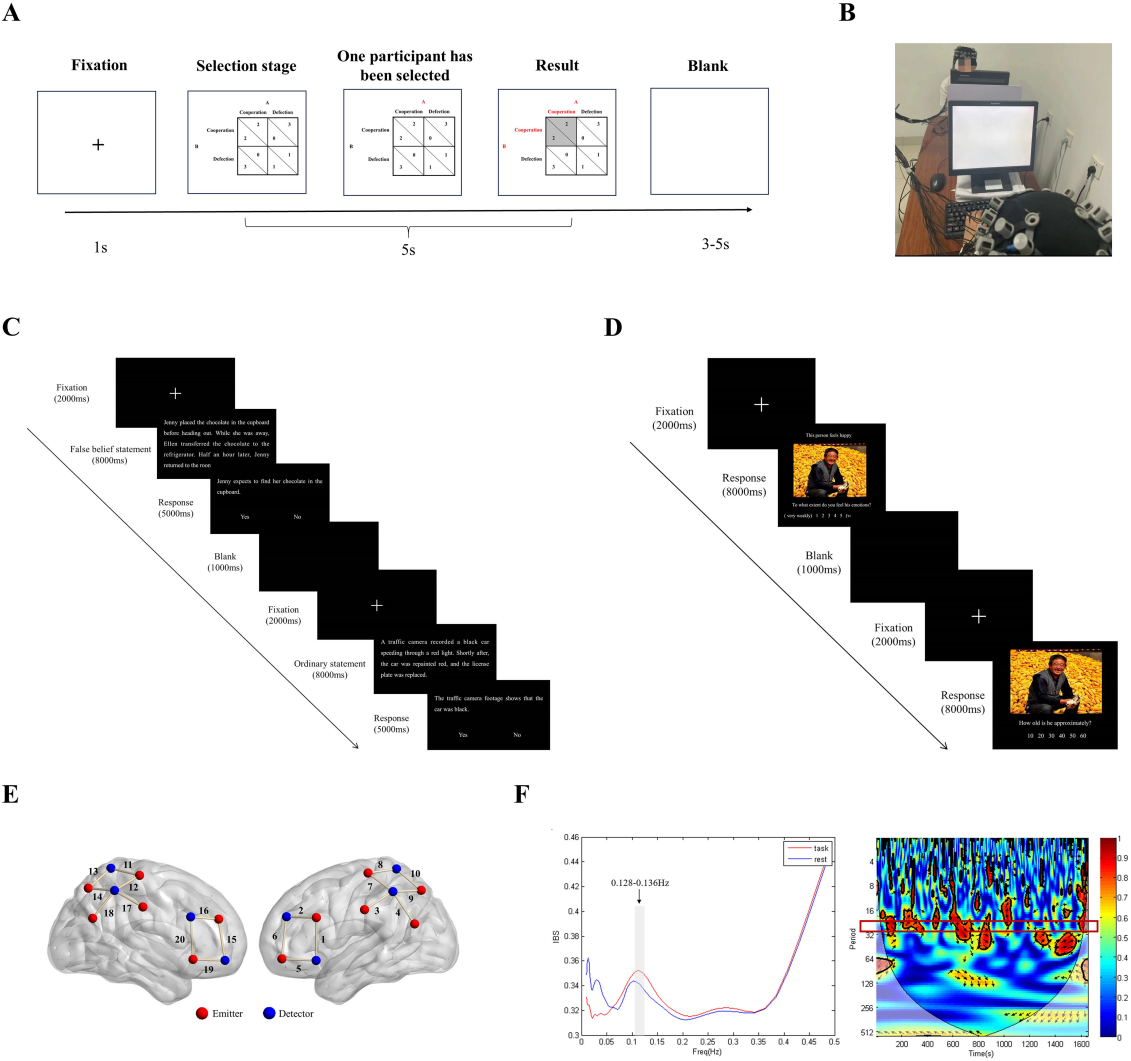
Experimental setting. **(A)** Procedure for the prisoner’s dilemma game task. **(B)** Illustration of the fNIRS hyperscanning experimental setup. **(C)** Procedure for the cognitive empathy task. **(D)** Procedure for the emotional empathy task. **(E)** Positions of the fNIRS channels. **(F)** Wavelet transform coherence (WTC) estimating IBS

Before the experiment, the experimenter explained the game rules to the participants, who then practiced for 10 trials. The participants were encouraged to maximize their gains. During the PDG, two participants sat facing each other, each in front of a computer screen, with a divider between them to prevent any information communication, such as eye contact (**Figure 1A**). A 2 × 2 payoff matrix appeared on the screen for 5 seconds, and participants chose either “cooperate” or “not cooperate” by pressing keys. Participant 1 used the D and F keys, whereas Participant 2 used the J and K keys. The key meanings (cooperate/not cooperate) were balanced across different participant dyads. When a participant selected, their score was highlighted in red. After both participants made their choices, the corresponding cell in the payoff matrix turned from white to gray. This entire process, from the appearance of the payoff matrix to the end of the result presentation, took 5 seconds. The jitter between trials was a random interval between 3-5 seconds. The detailed procedure is shown in **Figure 1B**. The game consisted of 120 trials, which were divided into three blocks of 6 minutes each, with a 2-minute rest between blocks.

After the cooperation task, the participants in the HL group continued to perform the cognitive empathy and emotional empathy tasks. In the cognitive empathy task, the first screen presented a fixation point, followed by a statement related to false beliefs. The participants then saw a relevant statement and had to judge its correctness. Afterward, a blank screen and another fixation point appeared, followed by an ordinary text and another relevant statement for the participants to judge. In the emotional empathy task, the first screen presented a fixation point, followed by an emotional image. The participants were asked to evaluate their level of empathy. Next, a blank screen and another fixation point appeared, followed by another emotional image, where participants assessed the age of the person in the image. The detailed procedures are shown in **Figures 1C, D**.

### fNIRS data acquisition and analysis

#### Data acquisition

A portable multichannel near-infrared optical imaging system (NirSmart, Danyang Huichuang Medical Equipment Co., Ltd., China) with wavelengths of 730 nm and 850 nm was used in this study. The data were sampled at a frequency of 11 Hz. The optical system comprised 24 emitters and 16 detectors, forming a total of 40 channels. For each participant, the optical system included 12 emitters and 8 detectors, creating 20 channels (the Brodmann areas and MNI coordinates can be found in Appendix 1). The average distance between the emitter and detector was 3 cm, with positions determined according to the international 10-20 system. Based on the Brodmann areas and MNI coordinates for each channel, the regions of interest and their corresponding channel sets in this study were as follows: bilateral IFG (8 channels: 1, 2, 5, 6, 15, 16, 19, and 20), bilateral IPL (8 channels: 7, 8, 9, 10, 11, 12, 13, and 14), and bilateral TPJ (6 channels: 3, 4, 9, 14, 17, and 18). The photopole position and channel position were identical for participants with low autistic traits and those with high autistic traits (see **Figure 1E**).

#### Data analysis

1. Individual brain activation data analysis. The NirSpark software (HuiChuang, China) package was used to preprocess the fNIRS signals, as in previous experiments (57). First, the raw light intensity signals were converted into an optical density curve, and then motion artifact interference was adjusted using the spline interpolation algorithm. Next, to decrease the noise caused by physiological fluctuations, such as heart rate and respiration, a bandpass filter with a cutoff frequency of 0.01-0.1 Hz was applied (57, 58). The optical density data were subsequently converted to relative changes in oxyhemoglobin (HbO) and deoxyhemoglobin (HbR) levels using the modified Beer-Lambert law. Since previous studies have shown that HbO signals are more sensitive to cerebral blood flow changes, the current study focused on HbO signals (14). The HbO concentrations for each block paradigm were superimposed and averaged to generate a block average result. Using a general linear model (GLM), the *β* values for the 20 channels of each participant were analyzed to reflect the level of brain activation.

2. IBS data analysis. The raw fNIRS data of each participant were preprocessed with the HOMER2 MATLAB package with the following processing workflow (59, 60). First, the original wavelength data were converted into optical density data. Second, principal component analysis (PCA) was performed to remove global physiological noise, and the covariance index was set to 80% (20, 61). The correlation-based signal improvement (CBSI) method was subsequently used to eliminate motion artifacts. Finally, the processed optical density data were converted into HbO and HbR concentration changes using the modified Beer–Lambert law. We focused solely on HbO signals, as they are a more sensitive indicator of changes in cerebral blood flow measured by fNIRS (14).

Preprocessed data collected during the resting phase, serving as the baseline, and task phase were entered into the IBS analysis. The IBS between two participants was computed using a wavelet transform coherence (WTC) algorithm. A MATLAB toolbox was used to calculate the WTC to detect consistent phase relationships between the time-frequency representations of the two-time series (62). We estimated whether IBS was greater during the task phase than at baseline using the WTC. IBS (averaged across channels in each dyad) was compared between the resting phase and the task phase using a series of paired sample *t* tests, one for each frequency band (frequency range: 0.01-0.5 Hz, cycle range: 2-100 s). This analysis yielded a series of *p* values that were false discovery rate (FDR)-corrected (*p* < 0.05) (63). The results indicated that the IBS of the task phase was significantly greater than that of the resting phase for the frequency range of 0.128-0.136 Hz. This range was selected as our frequency of interest (FOI). This range excludes the effects of physiological noise, such as heartbeats (0.8-2.5 Hz) and breathing (0.2-0.3 Hz). Therefore, the mean IBS of this FOI was selected for subsequent analyses. The right panel of **Figure 1F** displays the results for Channel 10 of participant group 1 in the LL group.

We computed the task-related IBS by subtracting the average IBS during the resting phase from that during the task phase. Fisher’s Z transformation was applied to the task-related IBS to generate a normal distribution (14). The resulting values for each channel were then assessed with 2 (group: HL vs. LL) × 2 (decision outcome: mutual cooperation vs. mutual defection) repeated-measures analysis of variance (ANOVA). The resulting *p* values were FDR-corrected for multiple comparisons (63).

## Results

### Individual behavioral results

#### Unilateral cooperation/defection rates

**Figure 2:**
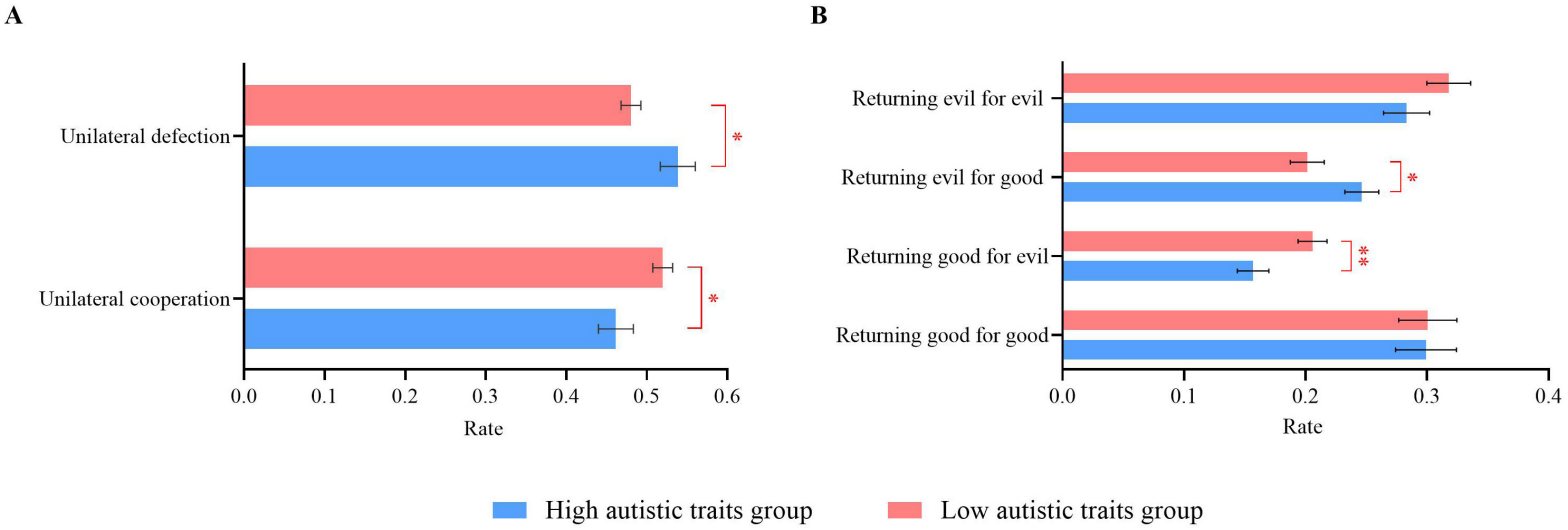
Individual behavioral results of high and low autistic traits groups during. **(A)** Unilateral cooperation/defection rates. **(B)** Different decision pattern rates. Error bars represent standard errors. ^*^ *p* < 0.05, ^**^ *p* < 0.01.

The unilateral cooperation rate was defined as the proportion of trials in which a participant chose “cooperate” out of 120 trials. Similarly, the unilateral defection rate was the proportion of trials in which a participant chose “not cooperate” out of 120 trials. To analyze the cooperation and defection rates, a 2 (autistic traits: high vs. low) × 2 (decision outcome: unilateral cooperation vs. unilateral defection) repeated-measures ANOVA was conducted. The interaction effect between autistic traits and decision outcome was significant (*F*(1, 110) = 5.39, *p* = 0.022, *η_p_* ^2^ = 0.05). Further simple effect analysis revealed that participants with high autistic traits had a significantly lower cooperation rate (0.46 ± 0.11) than did those with low autistic traits (0.52 ± 0.11, *p* = 0.022). Conversely, participants with high autistic traits had a significantly greater defection rate (0.54 ± 0.11) than did those with low autistic traits (0.48 ± 0.11, *p* = 0.037). The autistic traits and decision outcome main effects were not significant (*p*s ≥ 0.457), as shown in **Figure 2A**.

#### Impact of decision outcome on participants’ current decisions

In the PDG, the outcome of the opponent’s previous decision can influence the participant’s current decision. This influence can manifest in four patterns: returning good for good (cooperating after the opponent cooperates), returning good for evil (cooperating even after the opponent defects), returning evil for evil (defecting after the opponent defects), and returning evil for good (defecting after the opponent cooperates). To determine whether the impact of the opponent’s previous decision on the current decision varied between participants with high and low autistic traits, a 2 (autistic traits: high vs. low) × 4 (decision patterns: returning good for good vs. returning good for evil vs. returning evil for evil vs. returning evil for good) repeated-measures ANOVA was conducted. This analysis determined the occurrence rate of the four decision patterns in both groups.

The interaction effect between autistic traits and decision patterns was significant (*F*(3, 52) = 7.21, *p* < 0.001, *η_p_* ^2^ = 0.29). Further simple effect analysis revealed that the probability of returning good for evil was significantly greater in participants with low autistic traits (*M* = 0.21 ± 0.07) than in those with high autistic traits (*M* = 0.16 ± 0.06, *p* = 0.009). Conversely, the probability of returning evil for good was significantly greater in participants with high autistic traits (*M* = 0.25 ± 0.07) than in those with low autistic traits (*M* = 0.20 ± 0.07, *p* = 0.026).

The main effect of decision patterns was significant (*F*(3, 52) = 20.04, *p* < 0.001, *η_p_* ^2^ = 0.54). The rate of returning good for good (*M* = 0.30 ± 0.13) was significantly higher than that of both returning good for evil (*M* = 0.18 ± 0.07) and returning evil for good (*M* = 0.22 ± 0.08, *p*s ≤ 0.002). Additionally, the rate of returning evil for good (*M* = 0.22 ± 0.08) was significantly higher than the rate of returning good for evil (*M* = 0.18 ± 0.07, *p* < 0.001). The probability of returning evil for evil (*M* = 0.30 ± 0.10) was significantly greater than that of both returning good for evil (*M* = 0.18 ± 0.07) and returning evil for good (*M* = 0.22 ± 0.08, *p*s < 0.001). The main effect of autistic traits was not significant (*p* = 0.068), as shown in **Figure 2B**.

### Individual brain activation results

**Figure 3:**
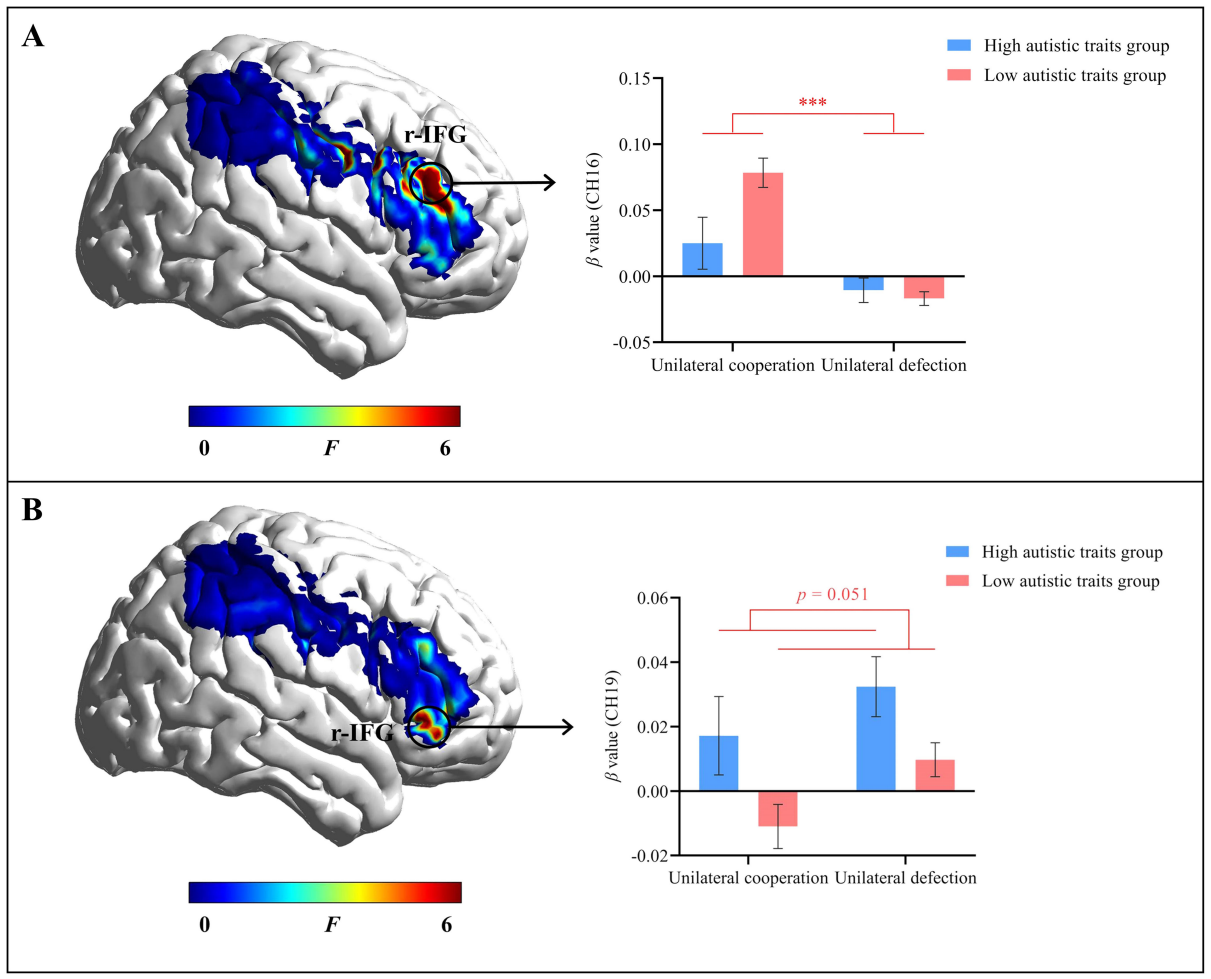
Individual brain activation results. **(A)** The main effect of decision outcome in channel 16 (r-IFG). **(B)** The main effect of autistic traits in channel 19 (r-IFG). Error bars represent standard errors. ^***^ *p* < 0.001.

A series of repeated-measures ANOVAs were conducted on the IBS for all channels, with autistic traits (high vs. low) as a between-participant factor and decision outcome (unilateral cooperation vs. unilateral defection) as a within-participant factor. After FDR correction, the main effect of decision outcome in channel 16 (r-IFG) was significant (*F*(1, 110) = 26.11, corrected *p* < 0.001, *η_p_* ^2^ = 0.19). The *β* value for unilateral cooperation (*M* = 0.07 ± 0.10) was significantly greater than that for unilateral defection (*M* = -0.02 ± 0.05), as shown in **Figure 3A**. The main effect of autistic traits on channel 19 (r-IFG) was marginally significant (*F*(1, 110) = 9.54, corrected *p* = 0.051, *η_p_* ^2^ = 0.08). The participants with high autistic traits had a significantly greater *β* value (*M* = 0.03 ± 0.06) than did those with low autistic traits (*M* = -0.001 ± 0.06), as shown in **Figure 3B**.

### Dyadic behavior results

**Figure 4:**
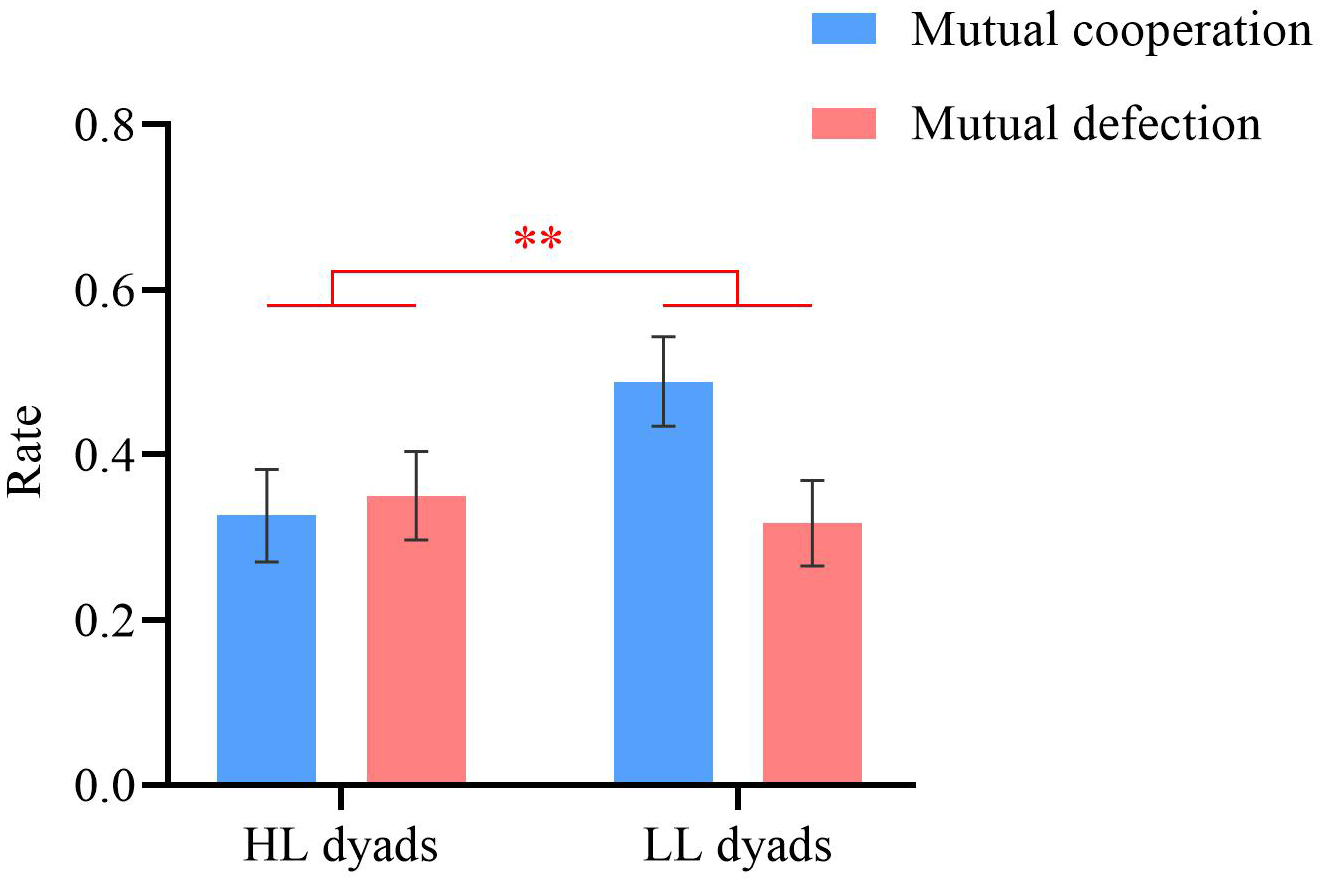
Mutual cooperation/defection rates. Error bars represent standard errors. ^**^ *p* < 0.01.

Repeated-measures ANOVAs were conducted on the mutual cooperation rate (i.e., the proportion of trials where both participants cooperate) and the mutual defection rate (i.e., the proportion of trials where both participants defect), with group (HL vs. LL) as a between-participant factor and decision outcome (mutual cooperation vs. mutual defection) as a within-participant factor. A significant main effect of group was observed (*F* (1, 54) = 9.62, *p* = 0.003, *η_p_* ^2^ = 0.15). The mutual cooperation and defection rates for the LL group (*M* = 0.40 ± 0.28) were significantly greater than those for the HL group (*M* = 0.34 ± 0.30). The main effect of decision outcome and the interaction effect were not significant (*p*s ≥ 0.187), as shown in **Figure 4**.

### Interpersonal brain synchronization results

A series of repeated-measures ANOVAs were conducted on the IBS for all channels, with group (HL vs. LL) as a between-participant factor and decision outcome (mutual cooperation vs. mutual defection) as a within-participant factor.

**Figure 5:**
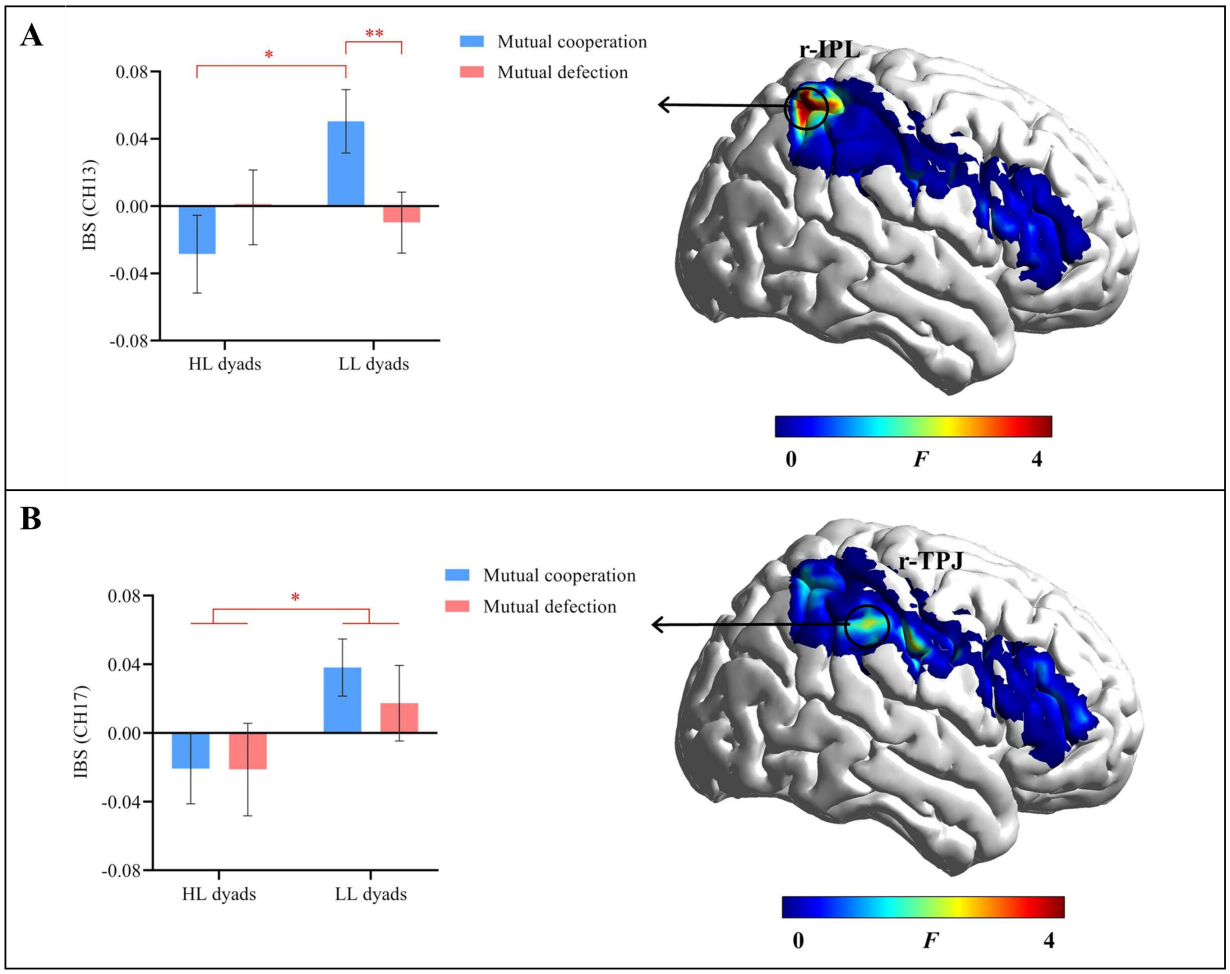
Results of IBS. **(A)** The interaction effect in channel 13 (r-IPL). **(B)** The main effect of group in channel 17 (r-TPJ). Error bars represent standard errors. ^*^ *p* < 0.05, ^**^ *p* < 0.01.

The results indicated a significant interaction effect in channel 13 (r-IPL) (*F*(1, 43) = 6.98, uncorrected *p* = 0.011, corrected *p* = 0.229, *η_p_* ^2^ = 0.14). Further simple effects analysis revealed that in the mutual cooperation condition, the IBS was significantly lower in the HL group (*M* = -0.03 ± 0.08) than in the LL group (*M* = 0.05 ± 0.11, *p* = 0.011). However, in the mutual defection condition, there was no significant difference in IBS between the HL group (*M* = -0.001 ± 0.10) and the LL group (*M* = -0.01 ± 0.09) (*p* = 0.753). In the LL group, the IBS was significantly greater in the mutual cooperation condition (*M* = 0.05 ± 0.11) than in the mutual defection condition (*M* = -0.01 ± 0.09, *p* = 0.007). For the HL group, there was no significant difference in IBS between the mutual cooperation condition (*M* = -0.03 ± 0.08) and the mutual defection condition (*M* = -0.001 ± 0.10, *p* = 0.287) (**Figure 5A**).

The main effect of group in channel 17 (r-TPJ) was significant (*F*(1, 43) = 4.48, uncorrected *p* = 0.040, corrected *p* = 0.802, *η_p_* ^2^ = 0.09). The HL group exhibited a significantly lower IBS (*M* = -0.21 ± 0.09) than the LL group (*M* = 0.03 ± 0.11). The main effect of decision outcome and the interaction effect were not significant (*p*s ≥ 0.609) (**Figure 5B**).

### Correlations between dyadic decision outcomes and interpersonal brain synchronization

To examine the relationship between IBS under different decision outcomes (mutual cooperation vs. mutual defection) and mutual cooperation/defection rates in the HL and LL groups, a Pearson correlation analysis was conducted on channels 13 and 17.

**Figure 6:**
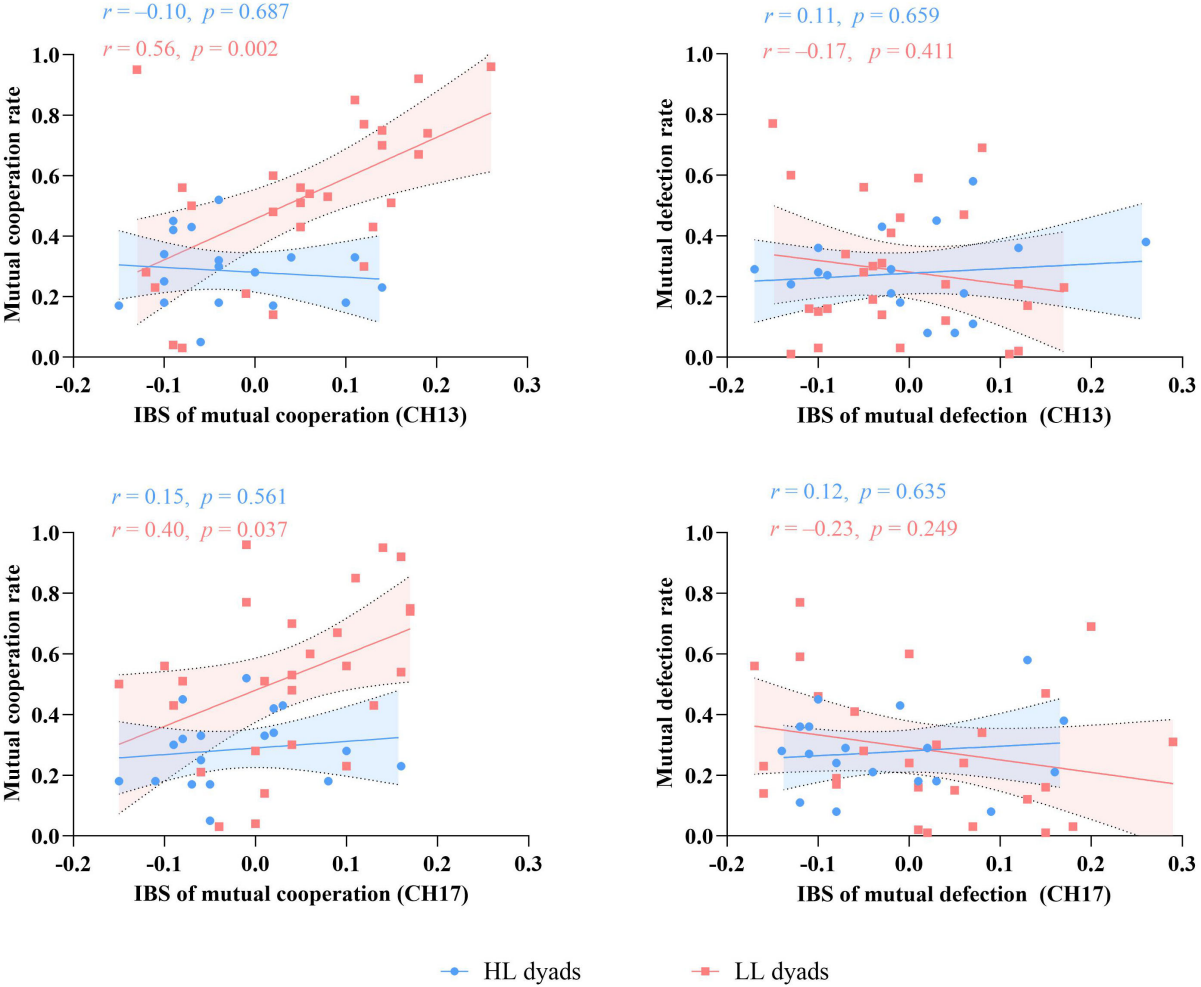
Correlation analysis of IBS in channels 13/17 with the mutual cooperation/defection rates in each group.

The results for channel 13 revealed a significant positive correlation between IBS and the mutual cooperation rate under the mutual cooperation condition for the LL group (*r* = 0.56, *p* = 0.002, 95% CI = [0.23, 0.78]). However, there was no significant correlation between IBS and the mutual defection rate under the mutual defection condition for the LL group or for the HL group under either condition. Similarly, in channel 17, the LL group exhibited a significant positive correlation between IBS and the mutual cooperation rate under the mutual cooperation condition (*r* = 0.40, *p* = 0.037, 95% CI = [0.03, 0.68]). No significant correlation was found between IBS and the mutual defection rate under the mutual defection condition for the LL group or for the HL group under either condition (**Figure 6**).

### Empathy results

Data from the empathy task were excluded for two participants who either did not answer seriously or failed to complete the task due to time constraints. The final analysis included 26 participants with high autistic traits and 26 participants with low autistic traits.

#### Cognitive empathy results

**Figure 7:**
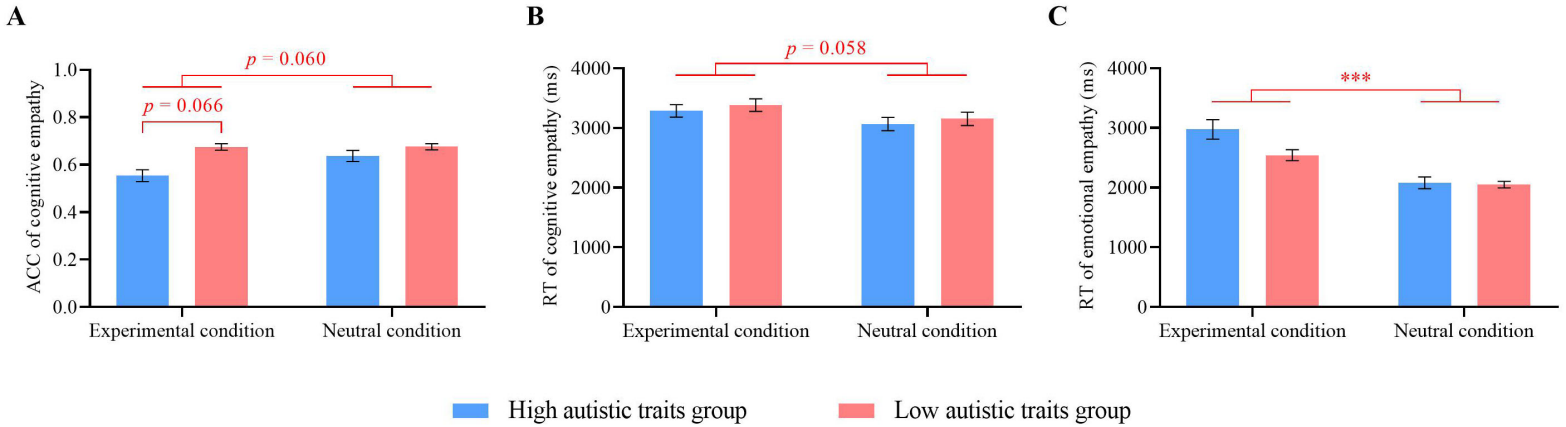
Empathy results. **(A)** Cognitive empathy accuracy. **(B)** Cognitive empathy response times. **(C)** Emotional empathy response times. Error bars represent standard errors. ^***^ *p* < 0.001.

Repeated-measures ANOVA was conducted on the cognitive empathy accuracy and response times, with autistic traits (high vs. low) as a between-participant factor and condition (experimental vs. neutral) as a within-participant factor. For cognitive empathy accuracy, the interaction effect between autistic traits and condition type was marginally significant (*F*(1, 50) = 3.52, *p* = 0.066, *η_p_* ^2^ = 0.07). Further simple effects analysis revealed that, under the experimental condition, participants with high autistic traits had lower accuracy (*M* = 0.56 ± 0.10) than did those with low autistic traits (*M* = 0.68 ± 0.12, *p* < 0.001). No significant difference in accuracy was observed between the two groups under the neutral condition. For participants with high autistic traits, accuracy in the experimental condition (*M* = 0.56 ± 0.10) was lower than that in the neutral condition (*M* = 0.64 ± 0.12, *p* = 0.010). No significant difference was found for participants with low autistic traits between the two conditions. There was a significant main effect of autistic traits (*F*(1, 50) = 10.69, *p* = 0.002, *η_p_* ^2^ = 0.18), with participants with low autistic traits showing greater accuracy (*M* = 0.68 ± 0.12) than those with high autistic traits (*M* = 0.60 ± 0.12). The main effect of condition was marginally significant (*F*(1, 50) = 3.71, *p* = 0.060, *η_p_* ^2^ = 0.07), with higher accuracy in the neutral condition (*M* = 0.66 ± 0.12) than in the experimental condition (*M* = 0.62 ± 0.12) (**Figure 7A**).

For cognitive empathy response times, the main effect of condition type was marginally significant (*F*(1, 50) = 3.77, *p* = 0.058, *η_p_* ^2^ = 0.07). The participants had longer response times in the experimental condition (*M* = 3335.39 ± 533.44 ms) than in the neutral condition (*M* = 3110.69 ± 563.55 ms). Neither the main effect of autistic traits nor the interaction effect was significant (*p*s ≥ 0.369) (**Figure 7B**).

#### Emotional empathy results

Repeated-measures ANOVA was conducted on emotional empathy accuracy and response times, with autistic traits (high vs. low) as a between-participant factor and condition (experimental vs. neutral) as a within-participant factor.

For emotional empathy accuracy, no significant differences were found across conditions. For emotional empathy response times, a significant main effect of conditions was observed (*F*(1, 50) = 32.75, *p* < 0.001, *η_p_* ^2^ = 0.40). The response times in the experimental condition (*M* = 2744.77 ± 760.98 ms) were significantly greater than those in the neutral condition (*M* = 2072.15 ± 527.36 ms). The main effect of autistic traits and the interaction effect were not significant (*p*s ≥ 0.106) (**Figure 7C**).

### Correlation analysis of empathy, unilateral cooperation rates, and individual brain activation

A Pearson correlation analysis was used to examine the relationships among participants’ scores in the cognitive/emotional empathy experimental conditions, their unilateral cooperation rates in the PDG, and *β* values in channels 16 and 19 under the unilateral cooperation condition. For participants with low autistic traits, cognitive empathy had a significant positive correlation with unilateral cooperation rates (*r* = 0.40, *p* = 0.043, 95% CI = [0.02, 0.68]) and a marginally significant positive correlation with the *β* value in channel 16 (r-IFG) (*r* = 0.38, *p* = 0.053, 95% CI = [-0.01, 0.67]) (**Table 2**). In contrast, for participants with high autistic traits, cognitive empathy had a marginally significant positive correlation with unilateral cooperation rates (*r* = 0.37, *p* = 0.064, 95% CI = [-0.02, 0.66]) and a marginally significant negative correlation with the *β* value in channel 19 (r-IFG) (*r* = -0.39, *p* = 0.052, 95% CI = [-0.67, 0.003]). Additionally, emotional empathy had a significant positive correlation with the *β* value in channel 19 (r-IFG) (*r* = 0.41, *p* = 0.039, 95% CI = [0.02, 0.69]) (**Table 3**).

**Table 2:** Correlation analysis of empathy, unilateral cooperation rates, and individual brain activation in participants with low autism traits.

	1	2	3	4	5
1. Cognitive empathy	1				
2. Emotional empathy	-.27	1			
3. Unilateral cooperation rates	.40^*^	-.07	1		
4. *β* value in channel 16	.38^†^	.17	-.07	1	
5. *β* value in channel 19	-.07	-.02	.15	.07	1

^*^ *p* < 0.05; ^†^ *p* < 0.07.

**Table 3:** Correlation analysis of empathy, unilateral cooperation rates, and individual brain activation in participants with high autism traits.

	1	2	3	4	5
1. Cognitive empathy	1				
2. Emotional empathy	-.27	1			
3. Unilateral cooperation rates	.37^†^	.16	1		
4. *β* value in channel 16	-.25	-.18	-.08	1	
5. *β* value in channel 19	-.39^†^	.41^*^	-.11	-.19	1

^*^ *p* < 0.05; ^†^ *p* < 0.07.

## Discussion

In this study, we employed fNIRS hyperscanning technology to investigate the cooperation rates of individuals with autistic traits. The results showed that individuals with high autistic traits exhibit lower cooperative abilities, both behaviorally and neurologically, than do those with low autistic traits. This reduced cooperation may be linked to their lower level of cognitive empathy.

The behavioral results revealed that the unilateral and mutual cooperation rates of individuals with high autistic traits were lower than those of individuals with low autistic traits. These findings validated our hypothesis and those of previous studies (7, 12), suggesting that individuals with high autistic traits are less likely to initiate or engage in cooperation. Additionally, individuals with high autistic traits were significantly more likely to choose a decision-making pattern of returning evil for good than were those with low autistic traits. The PDG used in this study is a socioeconomic decision-making task that encompasses both cooperation and defection decisions, in which individuals must decide whether to cooperate or defect during the task while also speculating on their partner’s potential choices (54). For an individual, choosing “defection” is the most rational choice; however, from the perspective of maximizing overall benefits, choosing “cooperation” can yield the highest total benefits for both parties (64). Thus, participants face a choice between a defection strategy that maximizes their own interests and a cooperation strategy that carries some risk but can maximize mutual benefits. Participants with high autistic traits tended to prefer returning evil for good as a means of maximizing their personal interests.

The individual brain activation results revealed that channel 16 (r-IFG) was activated during cooperative choices in individuals with both low and high autistic traits. These findings indicated that the r-IFG is closely associated with cooperation, which is consistent with the findings of previous neuroimaging studies (42, 46). However, under unilateral cooperation and defection conditions, individuals with high autistic traits exhibited significantly higher brain activation levels in channel 19 (r-IFG) than did individuals with low autistic traits. This result may be because the IFG is closely related to emotional and affective regulation (47). Actis-Grosso et al. (65) proposed the intense world theory, suggesting that individuals with high autistic traits experience excessive arousal in their perception, emotions, and memory when faced with intense emotional stimuli from others. This excessive arousal leads to strong anxiety, fear, and discomfort, prompting various avoidance behaviors to mitigate these feelings. Previous research has shown that brain regions associated with emotional sharing may cause discomfort under conditions of excessive arousal (66). Therefore, overactivation of the IFG in individuals with high autistic traits during interactions with peers may lead to avoidance behavior, limiting their degree of cooperation.

The IBS results revealed that the HL group had a significantly lower IBS in the r-TPJ than did the LL group, regardless of whether both parties were cooperating or defecting. Additionally, in the r-IPL, the HL group exhibited significantly lower IBS during mutual cooperation than did the LL group. These findings can be interpreted from both pathological and neurodiversity perspectives. From a pathological perspective, the TPJ and IPL are key components of the mirror neuron system, which plays a crucial role in understanding others’ behavioral intentions and is involved in social cognitive processes such as empathy and ToM (15, 41, 42, 45, 46). Successful cooperation relies heavily on the TPJ and IPL (17, 67). However, autistic individuals exhibit abnormalities in the mirror neuron system (68). For example, Balsa et al. (69) reported that, compared with non-autistic individuals, autistic individuals exhibited reduced dynamic functional connectivity in the TPJ, which was linked to increased social responsiveness scores. Similarly, May and Kana (70) reported that autistic individuals exhibited weaker activation in the IPL during executive function tasks. The insufficient IBS in the TPJ and IPL among individuals with high autistic traits, as found in this study, may be the neural mechanism underlying their cooperation difficulties.

The IBS results can also be interpreted from a neurodiversity perspective. Neurodiversity refers to the diversity of human thought, representing the infinite variations in neurocognitive function within our species (71). From this perspective, researchers have proposed the double empathy theory, which suggests that social deficits in autistic individuals may stem from a mismatch between autistic and non-autistic individuals (72, 73). In other words, autistic individuals may find it challenging to understand and communicate with non-autistic individuals; conversely, non-autistic individuals may have difficulty understanding the communication styles of autistic individuals (72, 74, 75). This issue affects both parties in the interaction, not just the autistic individuals. Crompton et al. (76) compared information transmission performance among the autistic group, non-autistic group, and mixed group. They reported no significant differences between the autistic group and the non-autistic group, but the mixed group exhibited a much greater decline in memory for details during information transmission. Similar findings have been reported in studies of individuals with high autistic traits. A recent study by Peng et al. (24), mentioned in the introduction, revealed effective communication within the HH group, but communication breakdowns occurred in the HL group due to the mismatch in neurotypes. Therefore, the insufficient IBS in the HL group in this study may have resulted from the inability of individuals with low autistic traits to understand the cooperative interaction styles of individuals with high autistic traits. This finding highlights the importance of mutual understanding between people with different neurotypes. In interpreting our findings, we lean more toward the pathological perspective, as it offers a direct explanation for the reduced IBS observed in the HL group. Specifically, the abnormalities in the mirror neuron system, particularly in the TPJ and IPL, are well-documented in individuals with high autistic traits and are associated with impairments in social cognition and cooperation, aligning with our results and providing a clear neurobiological mechanism for the observed cooperation difficulties (17, 67, 68).

Through experimental measurements, this study revealed that individuals with high autistic traits had significantly lower levels of cognitive empathy than those with low autistic traits. There was no significant difference in emotional empathy between the two groups, indicating that individuals with high autistic traits exhibit deficits specifically in cognitive empathy. This finding is consistent with the study’s hypothesis and previous research results (39), supporting the empathy imbalance theory of autism (38). Smith (38) posits that individuals with high autistic traits struggle to cognitively understand others’ emotions, leading to communication difficulties and a lack of awareness of how others perceive their behavior. These difficulties make them more likely to engage in unconventional actions. In this study, this was reflected in the lower cooperation rates and greater likelihood of returning evil for good among individuals with high autistic traits.

Correlation analysis among cognitive empathy, emotional empathy, unilateral cooperation rates, and individual activation results in the HL group revealed significant relationships. The cognitive empathy scores of individuals with low autistic traits were significantly positively correlated with individual cooperation rates and marginally positively correlated with activation in the r-IFG (channel 16). For individuals with high autistic traits, cognitive empathy scores were marginally positively correlated with unilateral cooperation rates and marginally negatively correlated with activation in the r-IFG (channel 19). This finding indicates a close relationship between cognitive empathy and cooperative behavior. Cognitive empathy involves recognizing and understanding others’ thoughts (29). In the PDG, if one person repeatedly chooses to cooperate, greater cognitive empathy can help the partner recognize that the first person is more focused on collective interests rather than personal interests, which may encourage them to choose cooperation. Xu et al. (27) reported that empathy for a partner in the PDG promotes cooperation but did not further distinguish whether cognitive or emotional empathy plays a key role. This study’s results emphasize the greater importance of cognitive empathy in cooperation.

Our findings revealed a significant positive correlation between emotional empathy scores and activation in the r-IFG (channel 19) in individuals with high autistic traits. However, there was no correlation between emotional empathy and unilateral cooperation rates. The IFG is associated with emotional regulation (47). Combining the behavioral results for empathy and the individual brain activation results for cooperation, our findings suggest that individuals with high autistic traits have intact emotional empathy and that their brain regions are more sensitive to activation. Smith (38) also posited that individuals with high autistic traits are highly sensitive to others’ expressed emotions but struggle to cognitively understand these emotions, making them more likely to avoid emotional interactions.

The absence of a correlation between emotional empathy and unilateral cooperation rates in this study may be related to the attributes of the PDG. In this task, decisions are influenced by the partner’s previous choices, allowing individuals to infer whether the partner prioritizes personal or collective interests, which involves cognitive empathy (77). Emotional empathy, on the other hand, involves the perception and sharing of emotions, typically conveyed through facial expressions or speech (29). In our experiment, participants sat face-to-face in front of computer screens, separated by a divider and without verbal communication. This setup limited their ability to perceive each other’s emotions, which explains the lack of observed correlation between emotional empathy and cooperation.

In summary, this study employed fNIRS hyperscanning technology to investigate how autistic traits influence cooperation and its neural mechanisms. We identified potential IBS neural markers of insufficient cooperation in individuals with high autistic traits, offering new perspectives for future research on cooperation and intervention in clinical autism patients. A recent study employing hypertranscranial electrical stimulation (hyper-tES) found that enhancing IBS was linked to improved behavioral coordination (78). Therefore, more research is needed to investigate the role of IBS in modulating cooperation, particularly in the r-IPL and r-TPJ, using noninvasive techniques such as transcranial magnetic stimulation and transcranial direct current stimulation. Additionally, through experimental tasks, this study further explored the relationship between empathy and cooperation in individuals with autistic traits by examining behavioral responses and individual brain activation. These findings provide insights into how empathy may influence cooperative behavior in patients with clinical autism.

## Limitations and future directions

Notably, this study had several limitations. First, using a dynamic IBS analysis method for interactive tasks would better reflect the process of cooperation. However, in the current study, the interaction time for each trial in the PDG is very short, and interactions under the same conditions do not recur consistently, making dynamic analysis inapplicable. Future research could employ longer-lasting cooperative interaction tasks to facilitate dynamic IBS analysis. Second, although this study revealed a direct relationship between cognitive empathy and cooperation, the specific role that cognitive empathy plays in influencing cooperation among individuals with autistic traits remains unclear. Future research could use experimental causal chain designs to further investigate the mechanisms through which cognitive empathy affects cooperation, such as whether it acts as a mediator.

## Conclusions

Autistic traits affect individual cooperation. Behaviorally, individuals with high autistic traits have lower unilateral and mutual cooperation rates than do those with low autistic traits. Neurologically, this is reflected in atypical individual brain activation patterns and lower IBS in individuals with high autistic traits. Moreover autistic traits influence cognitive empathy but do not affect emotional empathy. Additionally, there is a significant correlation between cognitive empathy and cooperation.

## Data Availability

The raw data supporting the conclusions of this article will be made available by the authors, without undue reservation.
