# Supplementary material for: Not on the same wavelength? How autistic traits influence cooperation: evidence from fNIRS hyperscanning
Source: Front Psychiatry. 2024 Dec 23;15:1514682. doi: 10.3389/fpsyt.2024.1514682 (PMC11701041; doi:10.3389/fpsyt.2024.1514682)
Supplement: Supplementary file 1 [file Table1.docx]

**Not on the same wavelength? How autistic traits influences cooperation: Evidence from fNIRS Hyperscanning**

**Supplementary material**

**Appendix 1**

**TABLE 1** The MNI coordinates and Brodmann regions of all channels.

| **Channel** | **MNI coordinate** | | | **BA** | **Anatomical label** | **Percentage of overlap** |
| --- | --- | --- | --- | --- | --- | --- |
|  | **x** | **y** | **z** |  |  |  |
| 1 | -60.54 | 23.57 | 8.35 | 45 | Pars triangularis Broca's area | 0.51 |
| 2 | -52.26 | 35.77 | 27.93 | 45 | Pars triangularis Broca's area | 1 |
| 3 | -67.01 | -35.16 | 43.26 | 40 | Supramarginal gyrus part of Wernicke's area | 0.74 |
| 4 | -63.21 | -56.35 | 36.61 | 39 | Angular gyrus part of Wernicke's area | 0.48 |
| 5 | -55.46 | 39.45 | -9.20 | 47 | Inferior prefrontal gyrus | 0.35 |
| 6 | -49.33 | 49.97 | 9.02 | 46 | Dorsolateral prefrontal cortex | 0.69 |
| 7 | -54.66 | -33.69 | 57.87 | 40 | Supramarginal gyrus part of Wernicke's area | 0.40 |
| 8 | -44.35 | -35.50 | 68.18 | 3 | Primary Somatosensory Cortex | 0.37 |
| 9 | -55.15 | -58.01 | 51.58 | 39 | Angular gyrus part of Wernicke's area | 0.46 |
|  |  |  |  | 40 | Supramarginal gyrus part of Wernicke's area | 0.54 |
| 10 | -41.72 | -58.39 | 60.44 | 40 | Supramarginal gyrus part of Wernicke's area | 0.51 |
| 11 | 44.94 | -34.10 | 68.28 | 3 | Primary Somatosensory Cortex | 0.44 |
| 12 | 56.27 | -33.91 | 56.97 | 40 | Supramarginal gyrus part of Wernicke's area | 0.43 |
| 13 | 41.31 | -56.52 | 62.66 | 40 | Supramarginal gyrus part of Wernicke's area | 0.52 |
| 14 | 54.39 | -56.69 | 52.90 | 39 | Angular gyrus part of Wernicke's area | 0.38 |
|  |  |  |  | 40 | Supramarginal gyrus part of Wernicke's area | 0.62 |
| 15 | 48.95 | 50.55 | 8.08 | 46 | Dorsolateral prefrontal cortex | 0.84 |
| 16 | 52.85 | 34.41 | 28.56 | 45 | pars triangularis Broca's area | 0.97 |
| 17 | 66.40 | -32.78 | 45.64 | 40 | Supramarginal gyrus part of Wernicke's area | 0.68 |
| 18 | 62.67 | -54.45 | 40.55 | 40 | Supramarginal gyrus part of Wernicke's area | 0.57 |
| 19 | 55.01 | 40.43 | -9.29 | 47 | Inferior prefrontal gyrus | 0.41 |
| 20 | 60.73 | 24.29 | 10.21 | 45 | pars triangularis Broca's area | 0.60 |
